# Pressurized Solvent Extraction of *Paulownia* Bark Phenolics

**DOI:** 10.3390/molecules27010254

**Published:** 2021-12-31

**Authors:** Paula Rodríguez-Seoane, Beatriz Díaz-Reinoso, Herminia Domínguez

**Affiliations:** 1 Chemical Engineering Department, Universidade de Vigo, Edificio Politecnico, As Lagoas, 32004 Ourense, Spain; paurodriguez@uvigo.es; 2 CITI (Center of Research, Transfer and Innovation), Universidade de Vigo, Parque Tecnoloxico de Galicia, Rua Galicia N° 2, 32900 Ourense, Spain; bdreinoso@uvigo.es

**Keywords:** *Paulownia*, subcritical water, supercritical CO_2_, antioxidants, valorization

## Abstract

*Paulownia* bark is mostly utilized jointly with wood, but the possibility of a separate valorization through the pressurized extraction of bark bioactives has been assessed. Subcritical water extraction and supercritical CO_2_ extraction are green technologies allowing shorter times than conventional solvent extraction under atmospheric shaken conditions. Subcritical water extraction was carried out at temperatures ranging from 140 to 240 °C and supercritical CO_2_ extraction was performed at different pressures (10, 20 and 30 MPa), temperatures (35, 45 and 55 °C) and ethanol concentrations (0, 10 and 15% (*w*/*w*)). Subcritical water extraction under a non-isothermal operation during heating up to 160 °C (19 min) provided extraction yields up to 30%, and the extracts contained up to 7% total phenolics with an ABTS (2,2′-azino-bis(3-ethylbenzothiazoline-6-sulfonic acid)) radical scavenging capacity equivalent to 35% the activity of Trolox, whereas at 240 °C, the yield decreased to 20%, but the phenolic content reached 21%, and the antiradical activity was equivalent to 85% of Trolox. Supercritical CO_2_ extraction at 30 MPa, 45 °C and 30 min reached a global yield of 2% after 180 min of extraction, but the product showed very low antiradical capacity. Gallic acid, vanillic acid, vanillin and apigenin were the major phenolic compounds found in the extracts.

## 1. Introduction

The genus *Paulownia* has been used as a folk remedy, especially in traditional Chinese medicine, and more recently has been considered an interesting source of secondary metabolites, such as flavonoids, lignans, phenolic acids and terpenoids [1]. Different parts of the tree (bark, fruit, xylem, and leaves) have shown therapeutic properties, mainly due to antioxidant, anti-inflammatory, antimicrobial and anticancer activities [2,3,4,5,6,7].

It has been suggested that the phenolic components with antioxidant properties may be responsible for the observed activity in traditionally used bark extracts [5,6,8,9,10], and they can also be used as chemotaxonomic markers [6]. Antioxidants can protect from the damage caused by free radicals and can protect lipid-containing food and cosmetic stuffs from oxidation. Among other mechanisms, antioxidants can exert their effect by scavenging free radicals. Therefore, the search for natural compounds with antiradical properties can provide alternative efficient antioxidants, which could offer additional biological properties and could be used as therapeutics.

Different extraction and purification strategies have been proposed to selectively recover *P. tomentosa* bark bioactives. Solvent fractionation of a crude ethanolic extract using a successive liquid–liquid extraction in *n*-hexane, dichloromethane, ethyl acetate and *n*-butanol has been proposed to obtain compounds with antioxidant properties [10]. Ethyl acetate provided the most active radical scavenging fraction and contained glucodistylin, luteolin, ellagic acid, cistanoside F, campneoside II, isocampneoside II, verbascoside and isoverbascoside [10]. Flavonoids (such as naringenin and quercetin), phenolic acids, (such as cinnamic acid and gallic acid) and phenylpropanoid glycosides (such as cistanoside F, acteoside, isoacteoside, campneoside II and isocampneoside II) were chromatographically isolated from the *n*-butanol soluble fraction [6]. Apigenin derivatives have been identified in acetone extracts [3]. A methanol extract of *P. tomentosa* stem bark, with phenylethanoid glycosides verbascoside and isoverbascoside as the predominant compounds, showed anti-inflammatory effects in RAW 264.7 macrophages and in an acute lung injury murine model [7]. A methanolic *P. tomentosa* bark extract and the fractions obtained by sequential liquid–liquid extraction with *n*-hexane, chloroform and water and chromatographic separation showed antiviral activity [4].

However, the use of greener solvents is preferred to obtain natural bioactives. In addition, obtaining wood and bark extractives is highly interesting to propose the valorization of important chemicals for future biorefinery valorization schemes [11]. In this context, the application of more selective and environmentally friendly extraction techniques would offer additional value of the final products [12]. Pressurized solvent extraction has advantages in relation to the enhanced efficiency in terms of extraction time [13]. Subcritical water extraction (SWE) is an eco-friendly technology performed at temperatures in the range of 100–374 °C, corresponding with the boiling point and critical point of water, respectively. Pressure is switched in the range of 2–15 MPa to maintain the water in its liquid state [14,15]. Under these conditions, water presents unique properties (low dielectric constant and high ionic product) that favor the extraction of apolar compounds and the partial breakage of the polysaccharidic structures [16]. Supercritical fluid extraction (SCFE) is a green and clean (GLEAN) extractive technique that operates with a fluid at temperatures and pressures over the critical point, a state without distinct liquid and gaseous phases, while simultaneously possessing properties of both phases [17,18]. The most frequent solvent used in this extraction method is carbon dioxide (CO_2_), which presents low critical conditions (7.4 MPa, 32 °C). CO_2_ is relatively highly available at relatively low cost, it is non-toxic, non-flammable and a good solvent for compounds with low volatility and polarity. It can be highly selective by adequately selecting operational conditions. Recent studies, using wood or bark as the starting material, employed the sc-CO_2_ extraction to the enrichment of different compounds. In this context, Barbini et al. [19] extracted pine bark using subcritical and supercritical CO_2_. The first step allowed a selective enrichment of the extract with unsaturated fatty acids despite the low extraction yields obtained. After removing most of the lipophilic compounds, sc-CO_2_ using ethanol as a polar cosolvent facilitated the recovery of more polar compounds, such as phenolics.

The aim of this work is to explore the potential of pressurized solvent extraction with subcritical water and with supercritical carbon dioxide to obtain extracts from *Paulownia elongata x fortune* with antiradical properties.

## 2. Results and Discussion

### 2.1. Composition

The proximal composition of *Paulownia bark* is shown in Table 1. The saccharidic fraction, accounting for almost 50% of the dry weight, was the most abundant, with glucose being the major component. Ethanol extractives accounted for 21%, whereas protein and ash represented a lower fraction. Qi et al. [20] reported a similar ash content (2.8%) for *Paulownia tomentosa* bark, with 80% volatile matter content and 17% fixed carbon content.

### 2.2. Subcritical Water Extraction

**Figure 1:**
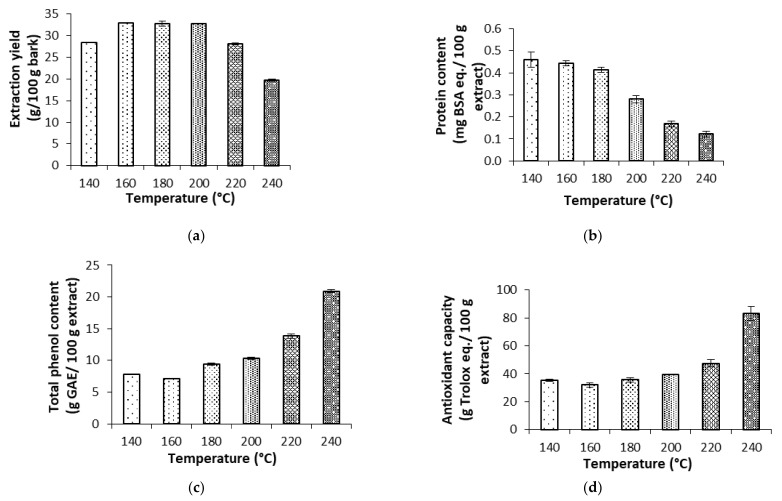
Influence of the final subcritical water extraction temperature on total extraction yield (**a**), protein content (**b**), the total phenolic content (**c**) and the antiradical capacity against ABTS (2,2′-azino-bis-(3-ethylbenzothiazoline-6-sulfonic acid)), (**d**), for the aqueous extracts from bark.

The influence of the final extraction temperature on the extraction yields is shown in Figure 1. A maximum of 33% was observed as a plateau region at 160–200 °C, with a steady decrease at higher values. However, the phenolic content showed a continuous increase in the range studied, more marked at the highest tested temperature, accounting for up to 21% of the dried extract. Lower solubilization can be attained with organic solvents, i.e., in three stages with methanol during 24 h [7].

According to data from Table 2, the saccharidic fraction was mostly found as oligomers, with glucose, arabinose and galactose as the most abundant. The operation temperature affected the different compounds differently; glucose in oligomeric units decreased with temperature, and the monomers only decreased at the highest values; galactose remained in oligomers up to 200 °C, but the monomer concentration decreased with temperature; and both xylose and arabinose content in oligomeric form increased with temperature, but the monomers were less affected. As expected, the organic acid concentration increased with operation temperature. Maximal extraction yields of 30% in the range of 160–200 °C were attained in subcritical water extraction, and at higher treatment temperatures, decreased values were observed until 20%. However, the phenolic content of the extracts increased more markedly with increasing temperatures, reaching more than 20%, and the antiradical capacity showed a similar trend. The ABTS radical scavenging capacity increased from 35% to 85% of the activity of Trolox. The protein content of the extract was under 0.5% and lowered with increasing extraction temperature.

### 2.3. Supercritical Fluid Extraction

**Figure 2:**
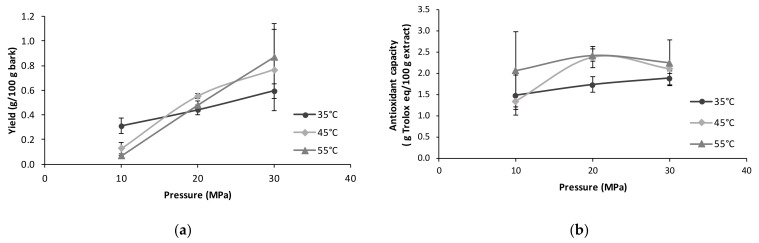
Influence of the extraction pressure and temperature on the extraction yield (**a**), and the antiradical capacity determined as ABTS (**b**) of *Paulownia elongata x fortunei* bark samples extracted with pure sc-CO_2_ for 30 min at the temperatures of 35 °C (dark gray), 45 °C (light gray) and 55 °C (intermediate gray).

Figure 2 shows the influence of pressure and temperature on the extraction yield and the antiradical properties of the sc-CO_2_ extracts from *P. elongata x fortunei* bark. As can be seen in Figure 2a, the use of higher pressures led to an improvement of extraction yield as a consequence of the increase in solubility caused by the supercritical CO_2_ density enhacement with pressure. Maximum extraction yields up to 0.8% were obtained when operating at the highest pressure. On the other hand, the effect of temperature on the extraction yield was the result of the dual effect that an increase in temperature may cause on the parameters, affecting solubility, i.e., the decrease in CO_2_ density and the increase in solute vapor pressure. In this work, the use of a lower temperature had a positive effect on the extraction yield during operation at the lower pressure, whereas at the higher pressure, a positive effect of temperature on the extraction yield was observed since the decrease in solvent density was compensated by the increase in the solute vapor pressure. This behavior, despite the important deviations observed in the experimental data at 30 MPa, suggests that the crossover point of the different isotherms could be in the range of 15–25 MPa. The ABTS radical scavenging test was selected for its simplicity, and since it is widely used, this assay can be valid for a rapid comparison of the bioactive potential of *Paulownia bark* extractives. The activity against ABTS radical was very low, under 2.5% Trolox equivalents, and no clear effect of temperature at the studied pressure conditions was found (Figure 2b).

**Figure 3:**
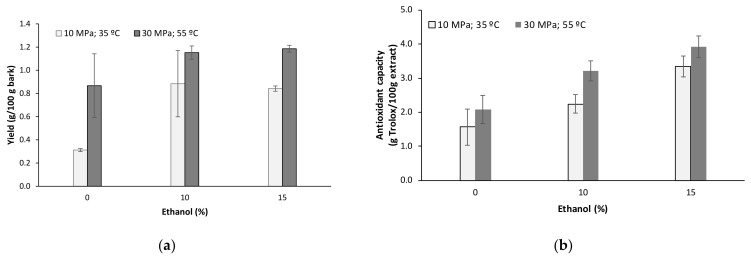
Influence of the ethanol concentration as a modifier on the extraction yield (**a**) and the antiradical properties, determined and TEAC value for ABTS scavenging capacity (**b**) of *Paulownia elongata x fortunei* bark at 10 MPa, 35 °C (white); and 30 MPa, 55 °C (gray) for 30 min.

The effect of the addition of ethanol as a polar modifier on the extraction yield and antioxidant activity of the extracts was evaluated (Figure 3). The use of ethanol as a solvent is highly frequent for its greener and renewable character, and in some cases offered higher yield than others, such as ethylacetate or water [12]. This influence was studied at 10 MPa and 35 °C and at 30 MPa and 55 °C, selected as the highest and lowest values of pressure operation, at temperatures providing higher yields and an extraction time of 30 min. At 10 MPa and 35 °C, the extraction yield increased significantly up to 0.8% with 10% ethanol as a modifier, whereas at 30 MPa and 55 °C, no significant effect of ethanol content on yield was observed after 30 min of extraction. Regarding the antioxidant capacity against the ABTS radical, a gradual increase with ethanol content was observed. The extraction yields were in the range of those reported for solvent fractions from a 95% ethanolic extract obtained at room temperature during 5 days, yielding 0.79% in the ethyl acetate fraction, which was also the most active radical scavenger. Other fractions represented lower yields—0.12% for the *n*-hexane and 0.10% for CH_2_Cl_2_, 0.01% for *n*-BuOH, whereas the water fraction represented 6.28% of the sample [10].

The extraction yield was higher than that of the fractions in dichloromethane, ethyl acetate or *n*-butanol fractions from a 95% ethanolic extract obtained at room temperature during 5 days from *P. tomentosa* [10]. The major compounds present in extracts were gallic acid, vanillic acid, vanillin and apigenin.

Supercritical fluid extraction of phenolic compounds from tree barks has been frequently reported and performed similarly to the present study. Bukhanko et al. [11] reported that supercritical carbon dioxide provided 2% yield from Norway spruce bark, a value slightly lower than from needles; the values were more than double using Soxhlet extraction. This technique proved suitable for the extraction of *Eucalyptus globulus* bark triterpenic acids [21] and *Pinus brutia* bark extractives 20 MPa, 60 °C and 3% ethanol [22]. Supercritical solvent extracts from *Eucalyptus globulus* bark obtained at 30 MPa, 70 °C, 20% ethanol and 10 g of CO_2_/min, with a yield of 0.48%, contained 6% gallic acid equivalents. Higher phenolic content could be found in ethanol:water extracts (15.9%) and with methanol:water (40.7%) [12].

**Figure 4:**
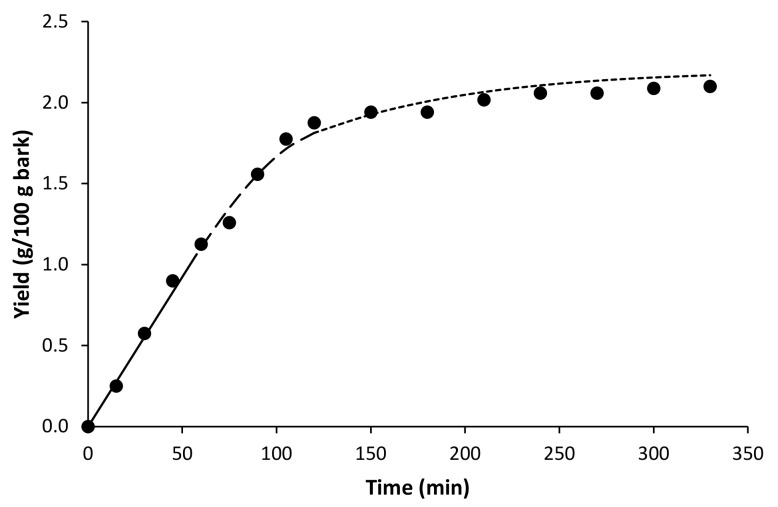
Kinetic extraction curve for the supercritical extraction of *Paulownia bark* at 30 MPa and 45 °C. Lines represents the BIC model: (–) CER period; (---) FER period; (···) DC period.

Kinetic experiments were carried out with CO_2_ at the pressure conditions that provided the highest extraction yields and at an intermediate temperature (30 MPa and 45 °C). The total extraction yield achieved after 330 min of extraction was 2%. The kinetic curves and their modeling offer information on the mechanism of the extraction process and on further scaling up. Among all the models proposed in the literature for fitting kinetic data, the BIC model [23] is the most widely used. Both experimental data and the fitted curve obtained with the broken-intact cell (BIC) model are shown in Figure 4. The parameters necessary for this model are presented in Table 3. The solubility of the solute (Ys) was initially estimated as the slope of the first part of the curve and then fitted to the experimental data along with the adjustable parameters of the model. The initial extractable solutes mass ratio in the raw material (x_0_) was fixed as the asymptotic value at infinite time.

The adjustable parameters of the model are also presented in Table 3. The experimental kinetic curve obtained can be divided in three periods controlled by different mass transfer mechanisms: (1) a constant extraction rate (CER), characterized by a fast extraction rate where the solutes present on the particle surface are transferred by convection to the solvent; (2) a falling extraction rate (FER), or transition period where the easily accessible solutes on the particle surface begin to be depleted and resistance to mass transfer at the fluid–solid interface starts to become significant; and (3) a diffusion-controlled period (DC), a slow extraction rate period where the mass transfer of the less accessible solutes is governed exclusively by diffusion. According to the results obtained by the model, the duration of the CER period was of 53 min, whereas the diffusion-controlled period (DC period) was not achieved until 120 min. The mass transfer coefficient in the solid phase, k_x_a, was three orders of magnitude lower than the mass transfer coefficient in the fluid phase, k_y_a, evidencing a strong limitation of solute mass transfer in the solid phase.

## 3. Materials and Methods

### 3.1. Raw Material

The hybrid *Paulownia elongata x fortunei* was harvested by Maderas Álvarez Oroza in Nois (Foz, Lugo, Galicia, Spain). Bark was collected in June 2017 and transported to the laboratory, where it was air dried at room temperature for 15 days, ground and stored in a dark and fresh place. Before drying, the average moisture content of bark was 72.13%.

### 3.2. Extraction

#### 3.2.1. Subcritical Water Extraction

**Figure 5:**
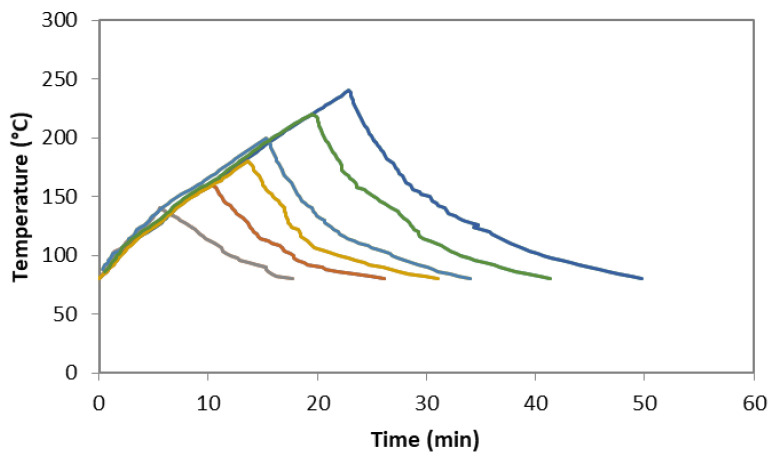
Heating profiles during non-isothermal treatments of *Paulownia bark*.

Subcritical water extraction under non-isothermal conditions was performed. Milled bark was mixed with distilled water at a liquid–solid mass ratio 7:1 (*w*/*w*). The reaction was carried out in a pressurized reactor (Parr Instrument Company, Moline, IL, USA) with 600 mL vessel capacity under constant stirring (150 rpm). Final heating temperatures in the range of 140–240 °C were studied, based on previous preliminary experiments. Once the selected temperature was reached, the system was cooled with water through a stainless steel coil located inside the vessel. Then, solid and liquid phases were separated by filtration. Operating with the highest heating rate, the temperature profiles at the operation temperatures, manually monitored, are shown in Figure 5.

#### 3.2.2. Supercritical Carbon Dioxide Extraction

Supercritical CO_2_ was performed in a supercritical fluid equipment (Thar Process, Inc., Pittsburg, PA, USA) with a 1000 mL extraction cell operating with a solvent mass flow of CO_2_ (99.99%, purity) fixed at 25 g/min. The amount of bark introduced into the reactor cell was 10 g with a moisture content of about 9–10%. The extractor was filled with glass balls until occupying the existing space. The study of influence of pressure and temperature was proposed. Experiments were performed at 10, 20 and 30 MPa and 35, 45 and 55 °C during 30 min. These conditions have been selected based on literature information [12,22] and preliminary experiments. Furthermore, the influence of absolute ethanol in concentrations of 10% and 15% (*w*/*w*) as a modifier was studied. Dynamic extractions were performed when the experimental conditions in the extractor were achieved. Extracts were recovered in a separator vessel with absolute ethanol.

Additionally, a kinetic curve was constructed during 330 min at 30 MPa and 45 °C, collecting the extract from the separator at intervals of 15–30 min. The modelling of the experimental data was carried out using the broken-intact cell model (BIC model) proposed by Sovová [23].

### 3.3. Analytical Methods

#### 3.3.1. Raw Material Characterization

The characterization of raw material was performed. For moisture determination (ISO 638 method), bark was dried in an oven at 105 °C for 24–48 h to reach a constant weight. Ash content was determined (ISO 776 method) after calcination at 575 °C for 6 h. The ethanol extractive content was gravimetrically determined after Soxhlet extraction. Total nitrogen was measured by elemental analysis (FlashEA 1112 Elemental analyzer, Thermo, Waltham, MA, USA) using 130 mL/min of He as a carrier gas and 100 mL/min as a reference gas. The oxygen flow was 250 mL/min, and the temperatures of the oxidation and reduction ovens were 900 °C and 680 °C, respectively. Protein content was determined by converting total nitrogen content using the factor 6.25.

Acid hydrolysis in two stages was performed before the chromatographic quantification of carbohydrates. In the first stage, the sample was digested with 72% sulfuric acid in a bath water at 30 °C for 1 h to break the polysaccharides. Then, the reaction was stopped by adding water. The second stage was performed with 4% sulfuric acid in an autoclave for 40 min in order to obtain monosaccharides. The liquid phase obtained was filtered through 0.45 µm cellulose acetate membranes and analyzed by HPLC using a refractive index detector (Model 1200, Agilent Technologies, Santa Clara, CA, USA). An Aminex HPX-87H (430150) column at 50 °C was used with 0.003 M H_2_SO_4_ at 0.6 mL/min as the mobile phase and an Aminex HPX-87P (424351) column at 80 °C operating with ultra-pure water at 0.4 mL/min as the mobile phase were used to determine different sugars. External standards for all the compounds were used.

#### 3.3.2. Extraction Yield

Extraction yield was gravimetrically determined. Aliquots of extract of known weight were dried in an oven at 105 °C until reaching a constant weight. Assays were carried out by triplicate.

#### 3.3.3. Total Phenolic Content and Antioxidant Profile

Total phenolic content (TPC) was determined by the Folin–Ciocalteu method proposed by Singleton and Rossi [24]. The results were expressed as grams of gallic acid equivalents (GAE). In this method, 0.5 mL extract or standard (gallic acid) and 3.75 mL distilled water were mixed. Then, 0.25 mL Folin–Ciocalteu reagent diluted 1:1 (*v*/*v*) and 0.50 mL sodium carbonate solution (10%, *w*/*v*) were also incorporated to the mixture. After one hour in the darkness, the absorbance of samples was measured at 765 nm. All assays were carried out by triplicate.

The phenolic compounds found in the extracts were analyzed using an Agilent HPLC 1100 instrument equipped with a Waters Spherisorb ODS-2 column (5 mm, 250 mm × 4.6 mm) and diode-array detector, operating at 30 °C, using 20 mL injection volume at 1 mL/min. Solvent A (acetonitrile/5% (*v*/*v*) formic acid in water, 10:90) and solvent B (acetonitrile/5% (*v*/*v*) formic acid in water, 90:10) were used in a non-linear gradient: 0 min, 100% A; 40 min, 85% A, 15% B; 45 min, 100% B; 55 min, 100% B; 60 min, 100% A; 65 min, 100% A. Identification was carried out by comparing the retention time and UV-visible spectral data with those of authentic compounds.

ABTS radical scavenging assay or a Trolox equivalent antioxidant capacity (TEAC) method was proposed by Re et al. [25] to obtain the ABTS (2,2′-azino-bis(3-ethylbenzothiazoline-6-sulfonic acid)) diammonium salt radical cation scavenging capacity of the above samples. To make 10 μL of extract or 6-hydroxy-2,5,7,8-tetramethylchroman-2-carboxylic acid standard (Trolox), 1.0 mL of diluted ABTS^·+^ solution was added. The ABTS^•+^ solution was obtained by the addition of phosphate buffer saline (PBS) (pH 7.4) to a 7 mM ABTS stock solution until reaching an absorbance of 0.70 at 734 nm. The mixture of the extracts with the ABTS^•+^ solution was measured at 734 nm after 6 min of incubation at 30 °C. A calibration curve with Trolox solutions was prepared following the same procedure described above, and the results were expressed as milligrams of Trolox equivalents. All assays were carried out by triplicate.

#### 3.3.4. Saccharidic Fraction of Hydrothermal Extracts

The saccharidic fraction of hydrothermal aqueous extracts were chromatographically analyzed. Monomers were obtained from direct analysis of the extracts, and before the oligosaccharide determination, a posthydrolysis process was necessary. Aliquots of the liquid extract were mixed with sulfuric acid to a final concentration of 4% and then were autoclaved for 20 min at 121 °C. The content of oligomers was determined by the difference between the content of monomers in the subcritical water extracts and in the posthydrolysis liquid phase. All samples were filtered through a cellulose acetate filter and analyzed by HPLC, following the same method used for carbohydrate determination described in Section 3.3.1.

### 3.4. Statistical Analysis

Significant differences between results were calculated by an analysis of variance (ANOVA) using the MINITAB 19 (Minitab Inc., State College, PA, USA) software. The significant differences (*p*  <  0.05) were evaluated by Tukey’s test. Mean values and their standard deviations were calculated and presented on the figure as error bars.

## 4. Conclusions

Two pressurized extraction methods were applied to *Paulownia bark*. Subcritical water extraction exhibited better results in terms of extraction yield and antiradical properties than sc-CO_2_ extraction. The maximum extraction yield was attained at intermediate temperatures of hydrothermal extraction (33%), whereas the total phenolic content and the antioxidant capacity rose with temperature and reached the highest values at 240 °C, exhibiting 20 g GAE/100 g extract and 85 g Trolox equivalents/100 g extract, respectively. Attending to the saccharidic fraction, the monomers of glucose and the oligomers of glucose, galactose and arabinose were the most abundant. On the other hand, supercritical fluid extraction yield (0.8%) was improved with pressure, being maximum at 30 MPa and 55 °C. The addition of ethanol as a polar modifier enhanced the extraction yield at 10 MPa and 35 °C. This positive influence of ethanol was also observed in the antioxidant capacity against the ABTS radical under both studied conditions. Further studies on the detailed characterization and potential applications are ongoing.

## Figures and Tables

**Table 1:** Composition of *Paulownia elongata x fortunei* bark.

Component	Content (%, d.w.)
Ash	3.00 ± 0.17
Ethanol extractives	21.32 ± 0.28
Protein	4.95 ± 0.51
Acid insoluble residue	25.62 ± 0.79
Carbohydrates	
Galacturonic acid	5.12 ± 0.13
Glucose	20.63 ± 0.15
Xylose	5.34 ± 0.30
Mannose	0.43 ± 0.11
Galactose	3.81 ± 0.09
Rhamnose	1.93 ± 0.16
Arabinose	1.83 ± 0.02

**Table 2:** Influence of the final extraction temperature on monomers (**a**) and oligomers (**b**) from saccharidic fraction of subcritical water extraction of *Paulownia bark*.

(a)						
Monomers (g/100 g Extract)	Temperature (°C)					
	140	160	180	200	220	240
Trehalose	7.5	6.07	5.29	2.93	-	-
Glucuronic acid	-	-	-	-	-	-
Galacturonic acid	-	-	-	-	-	-
Glucose	11.63	10.1	12.72	11.36	3.08	2
Xylose	0.7	0.51	0.21	0.25	1.29	0.87
Galactose	8.36	1.91	1.57	1.9	1.57	0.6
Rhamnose	0.22	0.15	0.65	2.55	1.29	0.5
Arabinose	-	0.08	0.84	3.2	1.42	0.92
Mannose	2.34	2.32	4.12	2.95	0.92	0.86
Mannitol	0.14	0.12	0.12	0.14	0.16	0.59
Formic acid	2.72	-	0.8	3.61	1.99	15.16
Acetic acid	-	0.27	0.61	2.36	8.2	20.57
(**b**)						
**Oligomers (g/100 g Extract)**	**Temperature (°C)**					
	140	160	180	200	220	240
Trehalose	-	-	-	-	-	-
Glucuronic acid	0.88	1.17	1.39	1	1.06	1.47
Galacturonic acid	8.41	11.57	5.65	1.48	0.05	0.07
Glucose	20.91	18.25	15.9	14.73	15.19	11.37
Xylose	0.7	0.37	1.26	9.07	16.15	1.25
Galactose	8.36	8.04	9.13	9.2	5.56	0.83
Rhamnose	6.72	6.69	6.83	5.13	1.76	-
Arabinose	3.24	13.01	17.74	13.28	1.8	-
Mannose	-	-	-	-	2.73	2.03
Formic acid	2.72	2.33	3.35	2.29	7.34	-
Acetic acid	0.94	0.88	1.34	2.97	3.88	-

**Table 3:** BIC Model Parameters Obtained for the sc-CO_2_ Extraction of *Paulownia bark* at 30 MPa and 45 °C.

Parameter	Value
k_x_a (min^−1^)	6.22 × 10^−3^
k_y_a (min^−1^)	1.95
r	0.74
Ys (kg/kg)	1.09 × 10^−4^
xo	0.022
t_CER_ (min)	53.3
t_FER_ (min)	117.7
AARD (%)	7.09

k_x_a: Mass transfer coefficient in the solid phase; k_y_a: mass transfer coefficient in the fluid phase; r: fraction of broken cells. Ys: solubility of the extract in the solvent; xo; initial solute mass ratio in the raw material; t_CER_; extration time at the end of the CER period; t_FER_: extraction time at the end of the FER period; AARD: absolute average relative deviation.

## Data Availability

Data are contained within the article.

## Sample Availability

Samples of the raw material and products obtained are available from the authors.
